# Recurrence, Microevolution, and Spatiotemporal Dynamics of *Legionella pneumophila* Sequence Type 1905, Portugal, 2014–2022

**DOI:** 10.3201/eid3005.231383

**Published:** 2024-05

**Authors:** Vera Manageiro, Vítor Borges, Raquel Rodrigues, Célia Bettencourt, Cecília Silva, João Paulo Gomes, Paulo Gonçalves

**Affiliations:** European Centre for Disease Prevention and Control, Stockholm, Sweden (V. Manageiro);; National Institute of Health Doutor Ricardo Jorge, Lisbon, Portugal (V. Manageiro, V. Borges, R. Rodrigues, C. Bettencourt, C. Silva, J.P. Gomes, P. Gonçalves);; Lusófona University, Lisbon (J.P. Gomes)

**Keywords:** Legionella pneumophila, ST1905, recurrence, outbreak, genomic epidemiology, bacteria, Portugal

## Abstract

We investigated molecular evolution and spatiotemporal dynamics of atypical *Legionella pneumophila* serogroup 1 sequence type 1905 and determined its long-term persistence and linkage to human disease in dispersed locations, far beyond the large 2014 outbreak epicenter in Portugal. Our finding highlights the need for public health interventions to prevent further disease spread.

*Legionella*, the causative agent of Legionnaires’ disease (LD), is a facultative intracellular gram-negative bacterium that is ubiquitous in freshwater environments. *Legionella* bacteria usually thrive in natural and artificial water sources, such as cooling towers, hot water tanks, and plumbing systems. Humans acquire infection by inhaling bacteria-contaminated droplets, usually generated by water systems or devices (*1*). Although the primary mode of transmission is exposure to contaminated aerosolized water, not all *Legionella* species known to cause disease in humans are exclusively associated with water sources. For example, infection with *L. longbeachae* is associated with exposure to soil and compost-related products (*2*). Although *Legionella* bacteria typically do not spread from person to person, that transmission route has been reported (*3*,*4*).

Incidence of *Legionella* infection in a population is primarily influenced by 3 factors: individual susceptibility, environment, and type of exposure. Outbreaks often occur in healthcare settings (e.g., hospitals or long-term care facilities) because of the presence of persons at increased risk for infection and severe disease (*5*).

One of the world’s largest outbreaks of LD (>400 cases and 14 deaths) occurred in the Vila Franca de Xira (VFX) region of Portugal, in 2014 (*6*,*7*). The outbreak was associated with the novel sequence type (ST) 1905 of *L. pneumophila* subspecies *fraseri* serogroup 1 strain (PtVFX/2014), which probably originated from a local industrial cooling tower (*6*,*7*). In-depth genomic analyses showed that PtVFX/2014 possesses a unique and mosaic genomic backbone marked by specific evolutionary and genetic traits (*3*), including a recently identified novel effector with nucleotropism (*8*), that may affect its ability to adapt and persist in diverse environments and cause human disease. The *L. pneumophila* ST1905 strain was also implicated by the strongest evidence to date of person-to-person transmission of LD (*3*,*4*). To our knowledge, *L.* *pneumophila* ST1905 has not been reported in countries other than Portugal. To elucidate the molecular evolution and spatiotemporal dynamics of *L. pneumophila* serogroup 1 ST1905 during 2014–2022, we investigated the phylogenetic relationship of strains isolated during that period at the National Institute of Health, Portugal, in the context of the National Legionnaires’ Disease Surveillance Programme.

## The Study

**Figure 1:**
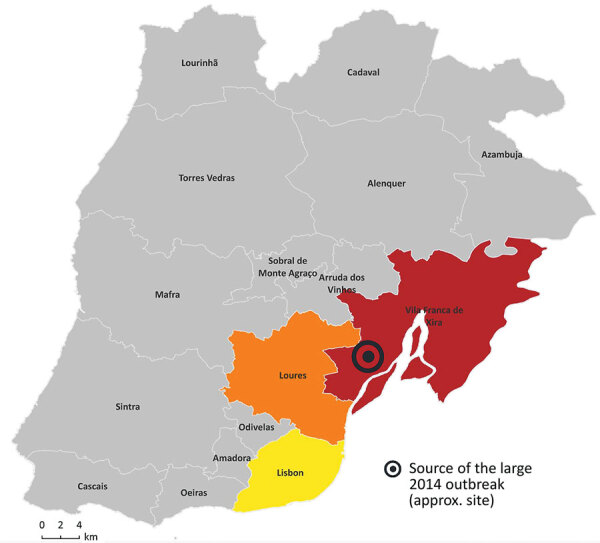
Geographic spread of 12 *Legionella pneumophila* sequence type 1905 isolates by probable municipality of exposure (clinical) or sampling (environmental), Lisbon region, Portugal, 2014–2022. Location of 2014 Legionnaires’ disease outbreak is indicated.

**Figure 2:**
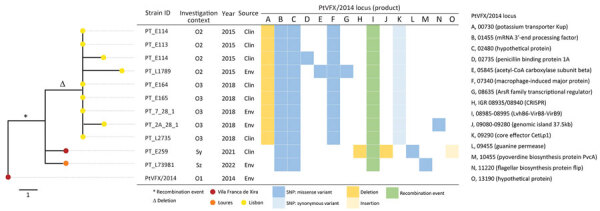
Core SNP-based phylogeny of whole-genome sequencing data from 12 *Legionella pneumophila* isolates obtained from samples collected in 3 interconnected municipalities in the Lisbon region, Portugal. Phylogeny was generated using a maximum-likelihood phylogenetic tree, rooted to the *L. pneumophila* PtVFX/2014 genome sequence (LORH00000000.1) (*2*). The investigation context, year, source, and genetic diversity profile within *L. pneumophila* genomes (SNPs, indels, and recombination event) compared with PtVFX/2014, are shown next to the tree. Clin, clinical; env, environmental; ID, identification; o, outbreak investigation; PtVFX, strain from 2014 Legionnaires’ disease outbreak in Vila Franca de Xira, Portugal; SNP, single-nucleotide polymorphism; s, sporadic case investigation.

Since the large 2014 LD outbreak, the ST1905 genotype has been identified several times in Portugal. For our study, we analyzed all *L. pneumophila* serogroup 1 ST1905 isolates (6 clinical and 6 environmental) obtained from samples collected in 3 interconnected municipalities in the Lisbon region (Lisbon, Loures, and VFX) in the context of clinical case investigations (outbreaks or as sporadic cases) (Figures 1, 2; Appendix 1; Appendix 2). We also included 2 culture-negative clinical samples genotyped as ST1905 for analysis of the temporal and geographic distribution of this strain after 2014 (Appendices 1, 2). 

Single-nucleotide polymorphism (SNP)–based diversity analysis confirmed that all ST1905 isolates were genetically similar to PtVFX/2014, differing by 3–6 SNPs. Overall, the observed microevolution across the 12 isolates was marked by 10 SNPs (9 nonsynonymous and 1 synonymous mutations), 4 insertion/deletions, and 1 small recombination event (Figure 2). The low number of SNPs during the 8-year period supports the notion that *L. pneumophila* evolves at a very slow rate, resulting in substantial temporal and spatial conservation, as previously reported (*9*,*10*). When compared with PtVFX/2014, all isolates presented a recombination event in an ≈2.5-kb region (contig 8, PtVFX/2014_08985-08995) belonging to the type IVA secretion system, which is associated with the survival of the bacteria in the environment (*11*). Of note, Lpn_PT_E259_y2021, isolated from a sporadic LD case, presented mutational events, including 2 deletions and 1 insertion, not found in any other isolates included in this study. The first deletion encompasses a 45,479-bp segment (contig 8, PtVFX/2014_09080-09280) that includes an ≈37.5-Kb genomic island containing an *lvh/lvr* type IVA secretion system cluster. That cluster was first identified within the *L. pneumophila* species in the PtVFX/2014 strain (PtVFX/2014_09115-09280), possibly because of interspecies transfer from *L. oakridgensis* (*3*). The second deletion spanned 142 bp and occurred in a CRISPR-associated intergenic region (PtVFX/2014_08935/08940). Moreover, we observed a frameshift 25-bp insertion at the PtVFX/2014_13190 locus, coding for a protein with unknown function. That locus seems to be specific for *L. pneumophila* subsp. *fraseri* and is located within a larger genomic region that contains known Dot/Icm substrates (*3*). Although we cannot make conclusions about a potential adaptive role of the observed mutations, it has been hypothesized that mutations found exclusively in clinical isolates, as in our study, might reflect human-specific adaptation (*10*). *L. pneumophila* can infect and replicate in human alveolar macrophages, but human-to-human transmission is assumed to be rare; thus, fixation of those mutations into *L. pneumophila* circulating in the human population is unlikely (*10*,*12*). Still, it has been proposed that the recent expansion of *L. pneumophila* in manmade water systems, together with the widespread distribution of specific clones at global scale, aligns with the potential dissemination between humans or from humans to the environment (*13*).

In-depth phylogenetic and microevolutionary analysis showed that ST1905 isolates did not cluster by year of isolation (Figure 2). Still, it is noteworthy that all clinical and environmental isolates associated with a particular location clustered apart, supporting persistence of the strain in that location and linkage between different events in some specific settings. Indeed, in one facility, located in the Lisbon municipality, we observed a genetic and epidemiologic correlation between the isolates collected in the outbreak investigation in 2015 and those from 2018 (Figures 1, 2; Appendix 1). In that facility, when both clinical and environmental isolates collected in the same investigation context were available, they differed by <3 SNPs, further supporting their epidemiologic linkage. Local microevolution is expected (*13*) and might contribute to fitness changes, such as increased tolerance to copper (*14*).

Three mutation events (2 SNPs and 1 recombination) were shared by all ST1905 isolates collected after the 2014 outbreak (Figure 2) We retrospectively inspected isolates from the 2014 outbreak for evidence of any of those mutations (*3*) and found that 1 nonsynonymous mutation (in PtVFX/2014_02480, coding for a hypothetical protein) had already been detected in 1 of the 2014 clinical strains (data not shown). That observation provides further evidence that the recurrent ST1905 detections derived from the large bacterial dispersion that occurred in 2014, probably resulting from atypical atmospheric conditions (*6*,*7*,*15*). It also supports the knowledge that *L. pneumophila* outbreaks can be caused by multiple same-strain subpopulations (present simultaneously in a source of infection and diversified over time) or even by different co-existing strains (*9*).

## Conclusions

Our results strongly indicate that the atypical *L. pneumophila* sg1 ST1905 strain is potentially persistent in diverse and geographically dispersed environments, far beyond the epicenter of the large 2014 outbreak in Portugal (VFX region). The recurrence of isolated cases or outbreaks in other regions with susceptible populations is thus of public health concern. Moreover, the observed microevolutionary traits and the potential of genetic recombination raise additional uncertainties regarding the evolutionary landscape of the ST1905 strain and its ability to further adapt and persist in the environment and, ultimately, cause human disease. Our study highlights the need for targeted public health preparedness and control strategies, emphasizing the added value of molecular epidemiology in the surveillance and management of LD.

Appendix 1 Isolate metadata information for study of recurrence, microevolution, and spatiotemporal dynamics of *Legionella pneumophila* ST1905, Portugal, 2014–2022.

Appendix 2 Additional information for study of recurrence, microevolution, and spatiotemporal dynamics of *Legionella pneumophila* ST1905, Portugal, 2014–2022.
