# Supplementary material for: Recurrence, Microevolution, and Spatiotemporal Dynamics of Legionella pneumophila Sequence Type 1905, Portugal, 2014–2022
Source: Emerg Infect Dis. 2024 May;30(5):1022–5. doi: 10.3201/eid3005.231383 (PMC11060437; doi:10.3201/eid3005.231383)
Supplement: Appendix 2 — Additional information for study of recurrence, microevolution, and spatiotemporal dynamics of Legionella pneumophila ST1905, Portugal, 2014–2022. [file 23-1383-Techapp-s2.pdf]

Article DOI: https://doi.org/10.3201/eid3005.231383

EID cannot ensure accessibility for supplementary materials supplied by authors. Readers who have difficulty accessing supplementary content should contact the authors for assistance.

Recurrence, Microevolution, and Spatiotemporal Dynamics of *Legionella pneumophila* Sequence Type 1905, Portugal, 2014–2022

Appendix 2

Materials and Methods

Selection of samples and isolates

This study includes all (n = 14) *L. pneumophila* samples and isolates collected after the large outbreak in 2014 that were identified as serogroup 1 and ST1905, in the context of the National Legionnaires' Disease Surveillance Programme, by the National Reference Laboratory for *Legionella*, in Lisbon, Portugal. In detail, it includes: 12 isolates (6 clinical and 6 environmental) and two positive specimens. A representative genome sequence of a ST1905 *L. pneumophila* PtVFX/2014 isolate collected during the 2014 outbreak, which was previously characterized (*1*), was included in this study as a reference. Details about all studied samples are available at the Appendix 1 Table 1 (https://wwwnc.cdc.gov/EID/article/30/5/23-1383-App1.xlsx).

DNA extraction and quantification

Isolation of bacterial DNA from 12 *L. pneumophila* isolates and from 2 *L. pneumophila* PCR-positive culture-negative samples was performed on the EMAG® platform (bioMérieux), an automated nucleic acid extraction platform based on magnetic silica technology. DNA was quantified by fluorescence spectroscopy using the Qubit Fluorometer (ThermoFisher Scientific).

L. pneumophila Sequence-Based Typing (SBT) and Whole Genome Sequencing (WGS)

SBT was performed at the National Reference Laboratory for *Legionella* using the DNA retrieved from pure cultures and uncultured clinical samples using the amplification of the 7 loci

in the SBT scheme, following the protocol of the European Working Group for *Legionella* infections (*2*). Sequencing libraries of DNA obtained from the *L. pneumophila* isolates (n = 12) was prepared using the Nextera XT DNA Sample Preparation Kit (Illumina), following manufacturer's instructions. Whole Genome Sequencing (WGS) was performed using Illumina MiSeq equipment (2x250 bp), as previously described (*1*).

Phylogeny and microevolutionary analysis

INNUca v4.2.2 (https://github.com/B-UMMI/INNUca) was applied to perform quality control of reads, draft de novo assembly and contigs quality assessment, and contamination detection. ReporType (*3*) (https://github.com/insapathogenomics/ReporType) was applied to perform *in silico* prediction of the *L. pneumophila* subspecies and serogroup from the draft assemblies. SBT profile ST1905 was also confirmed *in silico* through BLASTn (also using ReporType) against the SBT allele database available in https://github.com/tseemann/legsta. Snippy v4.6.0 (https://github.com/tseemann/snippy; settings:–mapqual 20–mincov 10–minfrac 0.51–basequal 20) was applied to the INNUca quality processed reads to perform reference-based mapping and SNP/indel analysis using the *L. pneumophila* PtVFX/2014 draft genome annotation (LORH00000000.1) as reference. Core genome SNP alignments were extracted from the Snippy-derived whole SNP alignment using ReporTree 1.1.2 (*4*). Whenever a mutation was reported for any of the studied isolates, the mapping files (BAM) of the other isolates were visually inspected in Integrative Genomics Viewer (IGV, https://igv.org/app/) to confirm/exclude its presence. Mapping files were also used to search and confirm the presence of indel events. A maximum phylogenetic likelihood tree was then constructed based on the curated core-genome SNP alignment (excluding SNPs found in ~2.5Kb recombination event found in reference contig 8, positions 8985–08995) using the Tamura 3-parameter evolutionary model in MEGA 11 (https://www.megasoftware.net/), incorporating 1000 random bootstrap replicates to assess node support within the tree.

References

1. Borges V, Nunes A, Sampaio DA, Vieira L, Machado J, Simões MJ, et al. *Legionella pneumophila* strain associated with the first evidence of person-to-person transmission of Legionnaires' disease: a unique mosaic genetic backbone. Sci Rep. 2016;6:26261. PubMed https://doi.org/10.1038/srep26261

2. Gaia V, Fry NK, Afshar B, Lück PC, Meugnier H, Etienne J, et al. Consensus sequence-based scheme for epidemiological typing of clinical and environmental isolates of *Legionella pneumophila.* J Clin Microbiol. 2005;43:2047–52. PubMed https://doi.org/10.1128/JCM.43.5.2047-2052.2005

3. Cruz H, Pinheiro M, Borges V. ReporType: a flexible bioinformatics tool for targeted loci screening and typing of infectious agents. Int J Mol Sci. 2024;25:3172. https://doi.org/10.3390/ijms25063172

4. Mixão V, Pinto M, Sobral D, Di Pasquale A, Gomes JP, Borges V. ReporTree: a surveillance-oriented tool to strengthen the linkage between pathogen genetic clusters and epidemiological data. Genome Med. 2023;15:43. PubMed https://doi.org/10.1186/s13073-023-01196-1
